# Four Parallel Pathways in T4 Ligase‐Catalyzed Repair of Nicked DNA with Diverse Bending Angles

**DOI:** 10.1002/advs.202401150

**Published:** 2024-04-06

**Authors:** Na Li, Jianbing Ma, Hang Fu, Zhiwei Yang, Chunhua Xu, Haihong Li, Yimin Zhao, Yizhen Zhao, Shuyu Chen, Lu Gou, Xinghua Zhang, Shengli Zhang, Ming Li, Ximiao Hou, Lei Zhang, Ying Lu

**Affiliations:** ^1^ MOE Key Laboratory for Nonequilibrium Synthesis and Modulation of Condensed Matter School of Physics Xi'an Jiaotong University Xi'an 710049 China; ^2^ Beijing National Laboratory for Condensed Matter Physics Institute of Physics Chinese Academy of Sciences Beijing 100190 China; ^3^ University of Chinese Academy of Sciences Beijing 100049 China; ^4^ Wenzhou Institute University of Chinese Academy of Sciences Wenzhou Zhejiang 325011 China; ^5^ College of Life Sciences Northwest A&F University Yangling 712100 China; ^6^ Hubei Key Laboratory of Cell Homeostasis College of Life Sciences Wuhan University Wuhan 430072 China; ^7^ Songshan Lake Materials Laboratory Dongguan Guangdong 523808 China

**Keywords:** conformational dynamics, parallel enzymatic pathways, protein machines, single molecules, T4 DNA ligase

## Abstract

The structural diversity of biological macromolecules in different environments contributes complexity to enzymological processes vital for cellular functions. Fluorescence resonance energy transfer and electron microscopy are used to investigate the enzymatic reaction of T4 DNA ligase catalyzing the ligation of nicked DNA. The data show that both the ligase–AMP complex and the ligase–AMP–DNA complex can have four conformations. This finding suggests the parallel occurrence of four ligation reaction pathways, each characterized by specific conformations of the ligase–AMP complex that persist in the ligase–AMP–DNA complex. Notably, these complexes have DNA bending angles of ≈0°, 20°, 60°, or 100°. The mechanism of parallel reactions challenges the conventional notion of simple sequential reaction steps occurring among multiple conformations. The results provide insights into the dynamic conformational changes and the versatile attributes of T4 DNA ligase and suggest that the parallel multiple reaction pathways may correspond to diverse T4 DNA ligase functions. This mechanism may potentially have evolved as an adaptive strategy across evolutionary history to navigate complex environments.

## Graphical abstract

Fluorescence resonance energy transfer and electron microscopy are used to unlock the intricacies of T4 DNA ligase catalysis of nicked DNA ligation. Four distinct conformations of both the ligase‐AMP and ligase‐AMP‐DNA complexes are discovered, suggesting parallel ligation pathways. These findings underscore the conformational versatility of T4 DNA ligase, suggesting multiple reaction pathways as adaptive strategies for DNA with diverse bending angles.

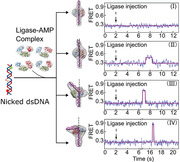

## 1 Introduction

Enzymes are ubiquitous across all living organisms, catalyzing chemical reactions within cells. They serve as structural scaffolds that recognize and bind their substrates with high efficiency, specificity, and selectivity.^[^ ^1^ ^]^ Motion and flexibility of enzyme structures have important roles in the formation of catalytically competent configurations for substrate recognition and product release.^[^ ^2^ ^]^ Enzymatic catalysis often involves small conformational changes that occur in tandem with chemical steps to stabilize the essential geometric alterations required for the chemical reactions.^[^ ^3^ ^]^ Consequently, diverse conformations for a given enzyme–substrate pair are considered an essential coordination mechanism for biomacromolecules, warranting thorough investigation.

DNA ligases play pivotal roles in linking DNA strands by catalyzing the formation of phosphodiester bonds between the 3′‐OH and 5′‐PO_4_ termini of DNA molecules. Ligation reactions generally involve three biochemical steps: i) adenosine 5′‐monophosphate (AMP) is sequentially transferred from ATP (or NAD^+^) to the active‐site lysine of a ligase, forming a covalent ligase–AMP intermediate; ii) the AMP is transferred to the 5′ phosphate of the nick site in the DNA, resulting in a covalent ligase–AMP–DNA intermediate; and iii) adenylation of the DNA activates the 5′ phosphate, facilitating nucleophilic attack by a 3′‐OH that displaces AMP and covalently connects the two ends at the nick site.^[^ ^4^ ^]^ ATP‐dependent ligases typically contain a nucleotidyltransferase domain that has an adenine nucleotide‐binding pocket and an oligonucleotide/oligosaccharide‐binding fold domain that consists primarily of a β‐barrel and one or more α‐helices that interact with the DNA minor groove.^[^ ^4^, ^5^ ^]^ Some DNA ligases may have additional domains such as a DNA‐binding domain^[^ ^6^ ^]^ or domains that interact with other proteins.^[^ ^6^, ^7^ ^]^ Prior to binding a nicked DNA, ligases tend to have open conformations.^[^ ^6b,c^ ^]^ Upon binding, the ligases form specific closed conformations to encircle the DNA through rigid‐body movements of the constituent domains,^[^ ^6^, ^8^ ^]^ thereby inducing local DNA bending and unwinding.^[^ ^4^, ^9^ ^]^

The T4 bacteriophage‐derived DNA ligase (hereafter referred to as T4 ligase) has a molecular weight of ≈55 kDa, and was the first ATP‐dependent ligase to be discovered. Consequently, DNA replication, recombination, and repair mechanisms of the T4 ligase have been extensively studied.^[^ ^4^, ^9^ ^]^ However, the recognition of DNA substrates that have different conformations and the ligation reaction pathways are still not clearly understood. To comprehensively understand the mechanisms of this enzyme, insights into its conformational ensemble structures are indispensable.^[^ ^10^ ^]^ Various techniques, including X‐ray crystallography and electron microscopy (EM), have been used to determine the structures of many biomacromolecules.^[^ ^11^ ^]^ However, real‐time observations of conformational changes and chemical reactions at biologically relevant time scales require techniques such as single‐molecule fluorescence resonant energy transfer (smFRET).^[^ ^12^ ^]^ By combining single‐molecule techniques with structure determination methods, dynamic processes can be accurately assigned to conformational snapshots.^[^ ^13^ ^]^

In this study, we investigated the dynamics of enzyme–substrate interactions using smFRET and EM. Our smFRET results demonstrate four distinct pathways during the ligation of nicked DNA. Our EM investigations show that the T4 ligase–AMP complex undergoes ATP hydrolysis, leading to the formation of four conformations. This process enables the selective binding of DNA substrates with various bending angles, ultimately leading to four distinct ligase–AMP–ligase conformations. Together with biochemical experiments, we demonstrate the existence of four parallel pathways in T4 ligase‐catalyzed repair of nicked DNA with diverse bending angles.

## 2 Results

### 2.1 Four Pathways for Repair of Nonsealable Nicked DNA by T4 Ligase

**Figure 1:**
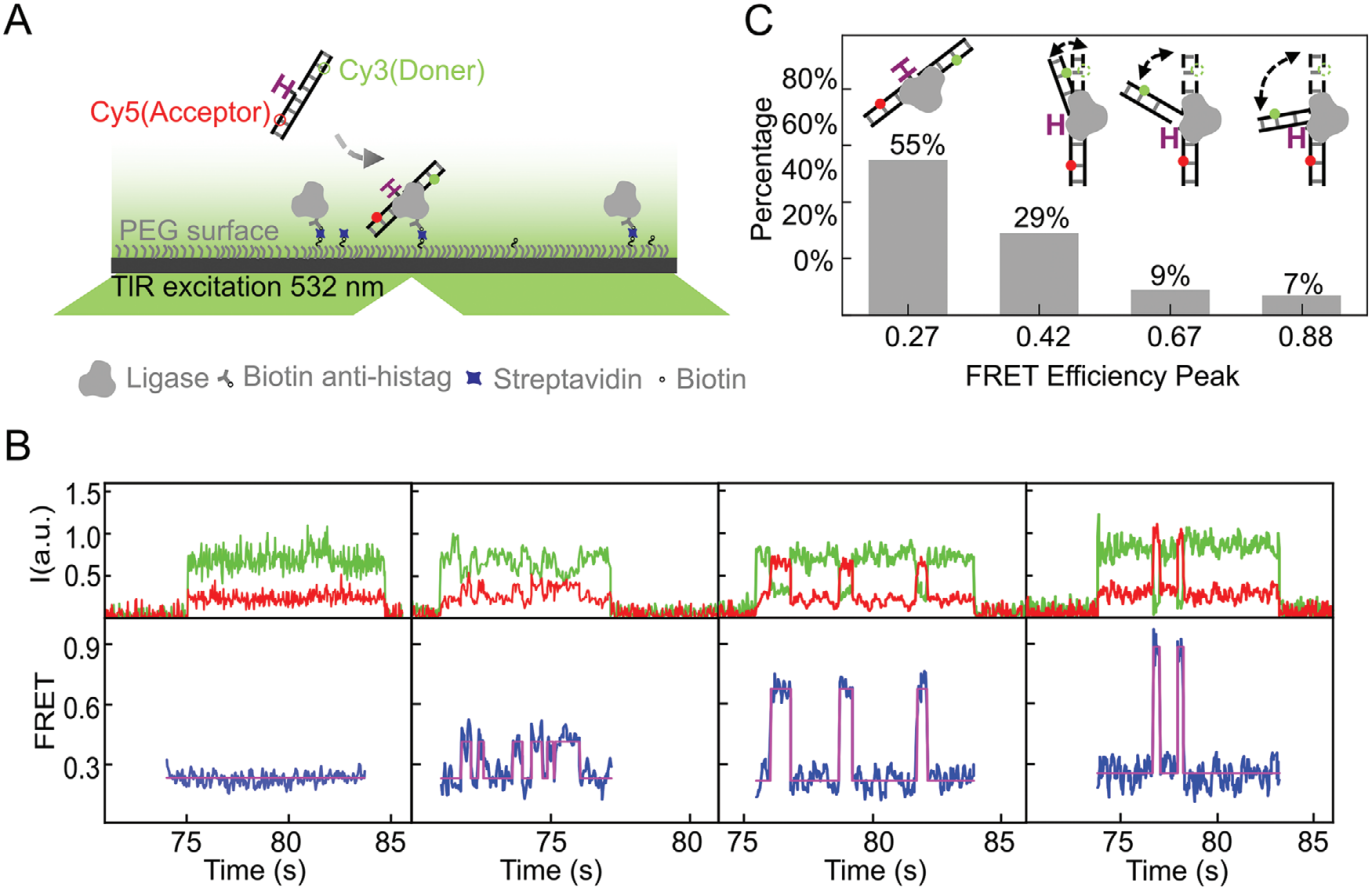
smFRET assay of nonsealable nicked DNA binding to surface‐bound T4 ligases. A) Schematic representation of the smFRET assay showing the use of 2′, 3′‐dideoxyribonucleotide (ddC, denoted as “H”) to block the final step of the nick‐sealing reaction. B) Typical time traces of the DNA conformational transitions. Green, donor intensities; red, acceptor intensities; blue, FRET values. C) Percentage distribution of DNA conformational transition patterns observed in the traces.

To investigate the dynamic processes involved in T4 ligase‐mediated ligation of nicked DNA, we performed smFRET on a total internal reflection fluorescence (TIRF) microscope. His6‐tagged T4 ligase molecules were immobilized on the surface of a reaction channel coated with anti‐His antibodies. We introduced a 3′‐strand terminated with 2′, 3′‐dideoxyribonucleotide (ddC) at the nick site to prevent the final step of the nick‐sealing reaction so that we could observe the repeated attempts of the surface‐bound ligases to ligate the nicked ddC DNA. Following the addition of 1 mm ATP, the DNA substrates were injected into the reaction channel for real‐time observation. Fluorescence signals were detectable only when the labeled DNA was in the illumination field (<200 nm near the surface, **Figure** **1**A) where they were captured by the surface‐bound ligases.^[^ ^14^ ^]^ The Cy3 and Cy5 dyes were labeled on the DNA, 21 base pairs (bp) apart (Figure 1A; Figures S1 and S2A, Supporting Information). This design ensured that the dyes did not come in contact with the ligases, thereby preventing interference to the enzyme and maintaining a low FRET efficiency of ≈0.27 before binding to the ligases (Figure S2A, Supporting Information). A low concentration (100 pM) of labeled DNA was used to minimize the fluorescent background, and therefore it took a few minutes for the nicked ddC DNA to diffuse to the ligases and produce detectable signals.

Approximately 55% of the TIRF signals had the lowest FRET value (0.27), indicating the straight DNA state was maintained throughout the reaction (pattern I in Figure 1B). The remaining signals had three distinct higher FRET values, detected using an unbiased step search algorithm.^[^ ^15^ ^]^ The higher FRET values were concentrated at peaks of 0.42, 0.67, and 0.88 (pattern II–IV in Figure 1B; Figure S2B, Supporting Information), indicating the presence of three concurrent nonstraight DNA states during the ligation process. Intriguingly, no transition among the three high FRET values was detected within a single time trace, with each high FRET value recurring separately (Figure 1B).

To further validate our findings, we conducted a control experiment (Figure S3, Supporting Information) by immobilizing the DNA on the surface of the reaction channel. Similar outcomes were observed after the introduction of T4 ligase. These results confirmed that upon binding to the nonsealable nick, the ligase repetitively attempted to repair the nick following one of the four observed patterns. We attributed these four patterns to four pathways through which the T4 ligase repairs nicks parallelly.

### 2.2 Four Pathways for Repair of Sealable Nicked DNA by T4 Ligase

**Figure 2:**
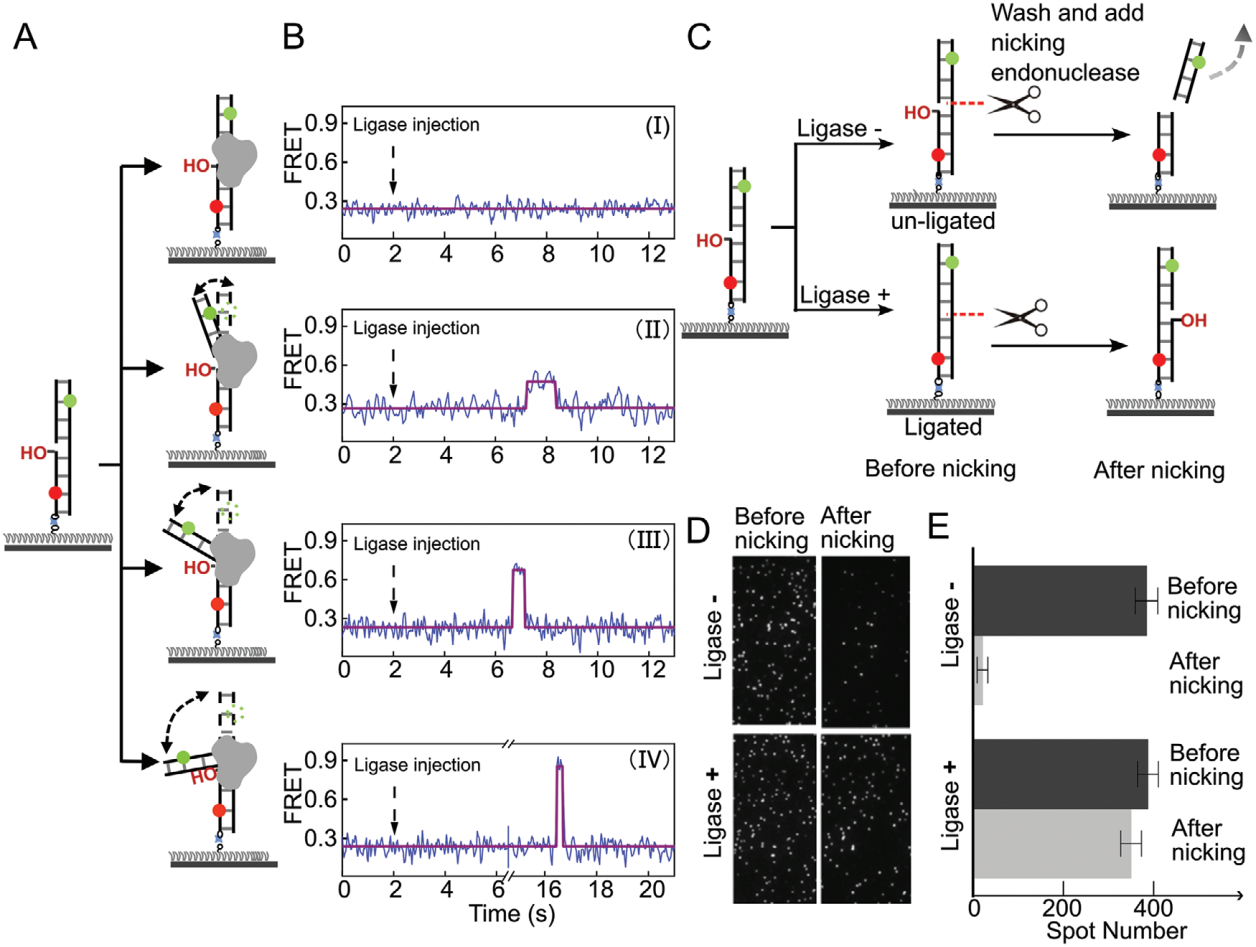
Reaction pathways in the T4 ligase‐catalyzed DNA repair. A) Schematic representation of the FRET assay with sealable nicked DNA. B) Typical FRET traces display the four signal patterns. C) Schematic illustration of the repair‐and‐then‐nicking assay. D) Representative micrographs from the repair‐and‐then‐cut assay. Micrographs before and after adding the nicking endonuclease (Nt.BsmAI) for 30 min are shown. Note that the images were taken at different locations on the slide surface. E) Histogram of the results of the single‐molecule repair‐and‐then‐nicking assay. The data are mean ± SD, *n* = 10.

In a canonical ligation process, a 5′‐phosphorylated strand permits the enzymatic transfer of AMP to its 5′‐terminus at the nick site of the DNA substrate. A 3′‐hydroxyl strand facilitates the completion of the final step in the nick‐sealing reaction. We immobilized sealable nicked DNA on the surface of a reaction channel. Four unique trace patterns were observed in parallel after 50 nm T4 ligase was injected and diffused to the surface‐bound nicked DNA (**Figure** **2**A). Among the time traces of the DNA conformational transitions, each high FRET value appeared only once in a single trace (Figure 2B). After a transition from the high to the low FRET values occurred, no further changes were detected. The results suggest that the DNA became straight after the ligation reaction regardless of whether the DNA was in a bent or straight state during the ligation process. These findings were independent of the nicked DNA sequence (Figure S4, Supporting Information). The TIRF signal was unchanged when double‐stranded DNA (dsDNA) without a nick was used in the experiments (Figure S5, Supporting Information), confirming that the high FRET values were induced by the ligation reactions.

Tethering the DNA on the surface (Figure 2A) allowed us to determine the proportion of the ligation product using a repair‐and‐then‐cut assay (Figure 2C). If DNA with a nick site is successfully repaired by the T4 ligase, the DNA will not be completely cleaved using only a nick‐making endonuclease to cut the opposite strand. However, if the opposite strand is cut very near an unrepaired nick site, the DNA will separate into two fragments. We validated this idea by performing ensemble assays (Figure S6, Supporting Information). In the smFRET assay, we incubated nicked DNA with T4 ligase for 1 min, then replaced the ligase with the nick‐making endonuclease. The assay showed that there was almost no decrease in the number of Cy5 spots. In a control assay where no T4 ligase was added, 94% of the Cy5 spots vanished (Figure 2D,E).

Together, our results indicate that the T4 ligase repaired the nicked DNA using one of the four patterns as illustrated in Figure 2B. This finding strongly suggests that four distinct reaction pathways occurred during T4 ligase‐catalyzed repair of nicked DNA.

### 2.3 Electron Microscopy Analysis of the Conformations of T4 Ligase and its Complexes

**Figure 3:**
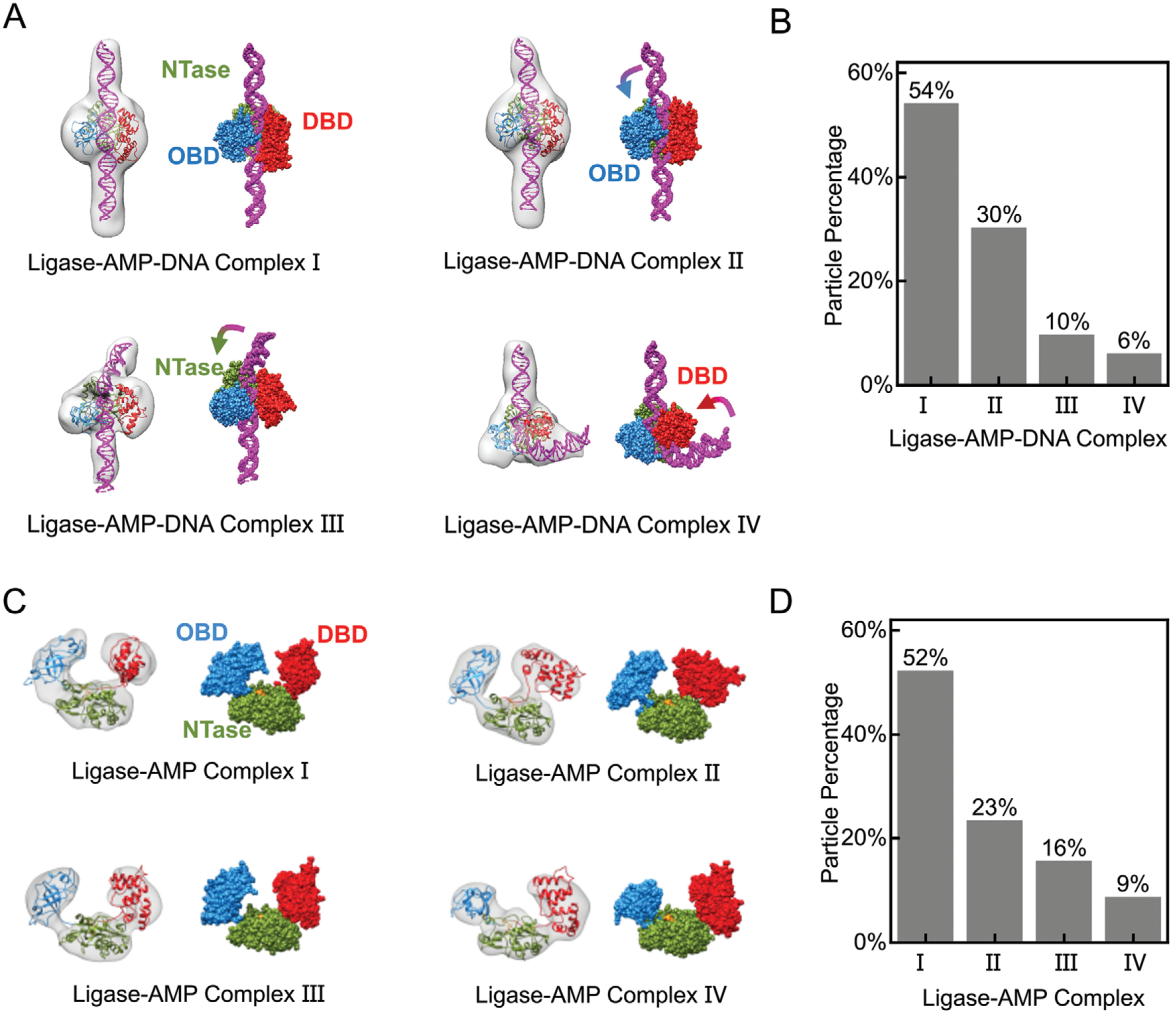
Four conformations of ligase–AMP–DNA complexes and four conformations of ligase–AMP complexes were obtained by negative‐staining electron microscopy. A) 3D density maps and refined structural coordinates of the four ligase–AMP–DNA conformations in which the DNAs are straight or bent. B) Histogram of the particle percentage of ligase–AMP–DNA complex conformations in A. C) 3D density maps and refined structural coordinates of the four ligase–AMP complexes. D) Histogram of the particle percentage of ligase–AMP complex conformations in (C). The density maps are Gaussian low‐pass filtered to 10 Å. Blue, oligonucleotide/oligosaccharide‐binding fold domain (OBD); red, DNA‐binding domain (DBD); green, nucleotidyltransferase (NTase) domain; orange, AMP; magenta, DNA. The gradient arrows indicate the direction of DNA bending.

To further investigate the conformations of the complexes detected in the smFRET assay, we used EM to analyze the 3D conformations of the T4 ligase complexes (Figure S7, Supporting Information). We used nicked ddC DNA of 144 bp (Table S1, Supporting Information) that consisted of an 18‐bp segment repeated eight times with the nicks serving as separators. Because the molecular weights were small (55–70 kDa), we used our previously reported negative‐staining (NS) protocol^[^ ^16^ ^]^ to image the T4 ligase–AMP–DNA complex. Both straight and bent DNAs with discretely attached ligases were observed (Figure S7A,B, Supporting Information). Based on reference‐free 2D classification, four major bending conformations characterized by DNA bending angles were identified (Figure S7C, Supporting Information). Subsequent 3D classification confirmed the presence of four main structures that corresponded to the major bending conformations, namely ligase–AMP–DNA complex I (DNA‐straight), and complexes II, III, and IV (DNA‐bent) (Figure S7D, Supporting Information). Postprocessing techniques were applied to improve the resolution, resulting in values of 13.4–14.6 Å (**Figure** **3**A; Figure S8, Supporting Information). The bending angles for ligase–AMP–DNA complexes II–IV were estimated to be ≈20°, 60°, and 100°, respectively, with particle percentages for complexes I–IV of ≈54%, 30%, 10%, and 6%, respectively (Figure 3B). Notably, the NS‐EM analysis of DNA‐free T4 ligase–AMP complex also detected four distinct conformations (ligase–AMP complexes I–IV) (Figure 3C; Figure S9, Supporting Information), with particle percentages of ≈52%, 23%, 16%, and 9%, respectively (Figure 3D), and resolutions of 9.2–12.9 Å (Figure S10, Supporting Information). Importantly, the distribution ratios of the four ligase–AMP complex conformations matched those of the four ligase–AMP–DNA complex conformations, as well as the trace percentages obtained from the FRET experiments for ligase–AMP–DNA complexes.

To refine the structural coordinates, we superimposed the crystal structure of T4 ligase complexed with a DNA intermediate (PDB ID: 6DT1)^[^ ^9b^ ^]^ and the nicked DNA onto the conformations constrained by data from the EM and FRET assays. We then conducted all‐atom molecular dynamics (MD) simulations (Figure S11, Supporting Information). In ligase–AMP–DNA complex I, the DNA had a linear conformation and, in ligase–AMP–DNA complexes II–IV, the DNA was bent and interacted with one of the T4 ligase domains (Figure 3A). In ligase–AMP–DNA complex II, the DNA bent toward the oligonucleotide/oligosaccharide‐binding fold domain (OBD) by ≈20°; in complex III, the DNA bent toward the nucleotidyltransferase (NTase) domain by ≈60°; and in complex IV, the DNA bent toward the DNA‐binding domain (DBD) by ≈100°. The orientation of the OBD in ligase–AMP–DNA complexes I and II allowed it to bind the nicked DNA along the minor groove,^[^ ^9b^ ^]^ whereas the DBD interacted with two regions of the minor groove.^[^ ^9b^ ^]^ The OBD and DBD orientations in these two conformations were consistent with those in the crystal structure. In ligase–AMP–DNA complex III, the DBD was directed along the major groove, and, in ligase–AMP–DNA complex IV, the OBD and DBD domains were both orientated almost vertically relative to their direction in ligase–AMP–DNA complexes I and II. The domain contours observed in ligase–AMP–DNA complexes I and II were similar to those found in the ligase–AMP–DNA complex of the cyanobacteria *Prochlorococcus marinus*,^[^ ^17^ ^]^ whereas ligase–AMP–DNA complex III was similar to the African swine fever virus complex.^[^ ^6c^ ^]^ Although large DNA bending angles in ligase–AMP–DNA complexes have not been reported so far, the DNA in NAD^+^‐dependent DNA ligase from *Thermus filiformis* may be bent to ≈90° when interacting with the NTase and helix‐hairpin‐helix domains.^[^ ^18^ ^]^ It is not possible to accurately calculate the bending angles using the FRET assay alone because both bending and twisting of the duplex DNA at the nick site contribute to changes in the FRET signals. Nevertheless, the four DNA bending conformations quantified by EM are in good agreement with our FRET results.

### 2.4 Selectivity of DNA Substrates with Different Bending Angles by the T4 DNA Ligase‐AMP Complex

**Figure 4:**
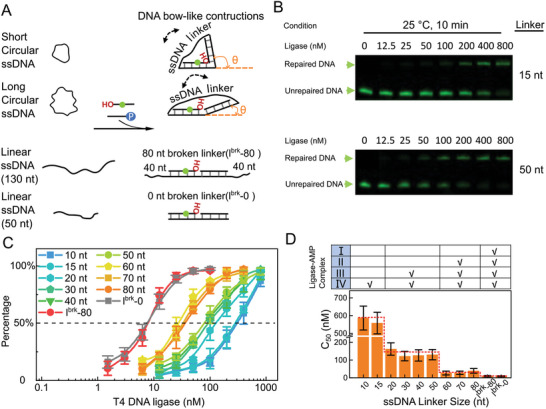
Ligation of nicked double‐stranded DNA (dsDNA) with different bending constraints. A) Schematic diagram of the bow‐like DNA constructions. In the constrained constructures, two 25‐nt single‐stranded DNA (ssDNA) fragments were annealed to a circular ssDNA. The bending of the nicked dsDNA is constrained by the unpaired ssDNA fragment. In the unconstrained constructures, two 25‐nt ssDNA fragments were annealed to a linear ssDNA. The two unconstrained samples are named l^brk^‐80 and l^brk^‐0 according to the length of the linear ssDNA. A Cy3 dye is labeled on one of the 25‐nt ssDNA strands. When nicked DNA is repaired, two 25 nt fragments will be ligated into one, which can be detected by denaturing PAGE/Urea gels. The phosphate groups are not shown in the annealed product cartoon. B) Ligation of the nicked DNA with a series of ligase concentrations at room temperature, as determined by 10% denaturing polyacrylamide gel electrophoresis. C) Percentage of the repaired DNA as a function of the ligase concentration. Error bars indicate standard deviation (*n* ≥ 3). D) Bar chart of the ligase concentration required to repair half of the nicked DNA (C_50_). The table on the top shows how the bending constraints affect the ligation efficiency. Error bars indicate standard deviation (*n* ≥ 3).

Our results imply that there is a correlation between the reaction pathways and the diversity of the ligase–AMP conformations. We investigated whether restricting the bending of the nicked DNA would affect the ligation efficiency. To this end, we engineered a series of nicked DNA with different bow‐like constructions in which the minimum bending angle of a 50‐bp nicked dsDNA was restricted by a ssDNA linker to the opposite ends of the dsDNA (**Figure** **4**A). A series of ssDNA linker lengths spanning 10–80 nucleotides (nt) was examined (Figure S12, Supporting Information). The shorter linkers impose strong restraint on the bending of the nicked DNA. After incubating the DNA constructures with T4 ligase for 10 min at 25 °C, the efficiency of the enzymatic reaction was evaluated by denaturing polyacrylamide gel electrophoresis (Figure 4B). The percentage of the repaired DNA as a function of the enzyme concentration was measured (Figure 4C). The ligase concentration required to repair half of the nicked DNA (C_50_) decreased rapidly as the linker lengths increased from 10 to 80 nt. Four plateaus can be clearly seen in the bar chart (Figure 4D).

These results suggest that the four ligase–AMP complexes observed in the EM assay (Figure 3C) may selectively bind their DNA substrates based on DNA bending angles. The ligase–AMP complex I conformation disfavored substrates with linkers ≈≤80 nt, which corresponds to bending angles ≥20° (see the table in Figure 4D). Similarly, the ligase–AMP complex II conformation disfavored substrates with linkers ≤50 nt (bending angles ≥60°), and the ligase–AMP complex III conformation disfavored substrates with linkers ≈≤15 nt (bending angles ≥100°). Consistent with this, DNA substrates with linkers ≈ ≤15 nt were repaired only by the ligase–AMP complex IV conformation. As a control, linear dsDNA substrates with no bending constraint (bars l^brk^‐0 and l^brk^‐80 in Figure 4D) can have almost unlimited bending, allowing all four ligase‐AMP complexes to repair them. Clearly, the enzymatic efficiency was restricted to only four values although the linker length could be from 10 nt to infinity. These results further confirm that the T4 ligase ligation reaction pathway does not engage the bending angles observed by EM in series, otherwise restricting DNA bending to a certain range would block the ligation.

## 3 Discussion

We have shown that the dsDNA in the T4 ligase–AMP–DNA complex can have four distinct conformations characterized by DNA bending angles of ≈ 0°, 20°, 60°, and 100°. Contrary to the conventional notion of sequential reaction steps of an enzyme with multiple conformations, we found that the T4 ligase catalytic process had four concurrent pathways, each correlating with a specific DNA bending angle. Our FRET assay aligned with the EM observations, showing that the T4 ligase has four ligase–AMP conformations that transit to four ligase–AMP–DNA conformations upon DNA engagement. We subsequently conducted a comprehensive exploration of ligation activity using DNA bow‐like constructions to substantially restrict the DNA bending. This strategic manipulation allowed the discernment of four discrete levels of ligation efficiency that were intricately linked to the DNA bending angles. Bent conformations of DNA appear to be intrinsically linked to the nick‐sensing process, representing a crucial structural adaptation around the catalytic site of the ligase.

Notably, this observation of multiple reaction pathways of an enzyme with one catalytic site is consistent with the principle of substrate selectivity. Together, our data suggest that the bending angle of nicked DNA is a pivotal feature that influences the enzymatic activity of T4 ligase. Enzymes usually modulate their inherent structures upon ligand binding, which enhances the catalytic efficiency across diverse substrates.^[^ ^19^ ^]^ This concept is supported by enzymology and structural biology principles.^[^ ^20^ ^]^ This polymorphism has enabled enzymes to fine‐tune substrate selection and adapt to diverse environments during evolution.^[^ ^21^ ^]^ T4 ligase exhibited selectivity for different DNA bending angles by forming four different ligase–AMP conformations. The ability of T4 ligase to repair diverse DNA structures can plausibly be attributed to its repertoire of multiple reaction pathways. Our findings provide a deep insight into the evolutionary adaptation of enzymes in simple organisms and have implications for higher organisms. This information may be particularly valuable in further research on the numerous ligases found in eukaryotic cells that have more complex functionalities and survival environments and serves as a reference for developing investigation frameworks and technique strategies.

## 4 Experimental Section

### T4 DNA Ligase Expression and Purification

The T4 DNA ligase (1–487, ≈55 kDa) without any tag was purchased from New England Biolabs Inc.(NEB), which was expressed and purified from *Escherichia coli* C600 pcl857 pPLc28 lig.^[^ ^22^ ^]^ For the His6‐tagged T4 DNA ligase, the gene encoding T4 DNA ligase was directly cloned from the T4 bacteriophage and then cloned into the pET15b vector with a 6×His tag fused at its N terminus. The His6‐tagged ligase was expressed in the *Escherichia coli* strain BL21 (DE3) and purified by FPLC with sequential chromatography on Ni‐NTA (GE Healthcare) and Superdex200 10/300 GL column (GE Healthcare). The final purified His6‐tagged ligase was determined to be >95% pure using sodium dodecyl sulfate‐polyacrylamide gel electrophoresis (SDS‐PAGE) and stored at −80 °C for further use.

### DNA Constructs

All oligonucleotides required to prepare the DNA substrates were purchased from Sangon Biotech Co., Ltd (Shanghai, China). The sequences are described in Table S1 (Supporting Information). DNA was annealed by incubating at 95 °C for 5 min and then slowly cooled down to room temperature for ≈7 h. Annealing was carried out in an annealing buffer containing 50 mm NaCl and 25 mm Tris‐HCl (pH 7.5 at 25 °C). For the circular ssDNAs (Figure S12, Supporting Information), each linear ssDNA (60, 65, 70, 80, 90, 100, 110, 120, 130 nt, Sangon Biotech Co., Ltd) was annealed with a short oligonucleotide (25 nt, Sangon Biotech Co., Ltd) complementary to both ends of the longer linear ssDNA, resulting in precircular DNA. Then, the pre‐circular DNA was ligated (4 °C for 16 h) using T4 DNA ligase (NEB). The products were separated with the denaturing 10% PAGE gel electrophoresis. Digestion of linear ssDNA by Exonuclease I(EXO I) (NEB) before electrophoresis separation was optional. All circular ssDNA molecules passed the EXO I assay and were stored in 10 mm Tris‐HCl (pH 8.0) buffer at −20 °C for later use. When the circular DNA anneals with two 25 nt fragments to form bow‐like DNA, there is a significant difference in mechanical properties between the dsDNA and ssDNA, leading to an approximation of a triangular structure. For the bare bow‐like DNA, based on previous experimental measurements^[^ ^23^ ^]^ and the ssDNA length‐force curve,^[^ ^24^ ^]^ we estimated a range of bending angles constrained by the 10–80 nt linkers, approximately ranging from 10° to 130°.

### Buffers

The reaction buffer contains 1 mm ATP, 50 mm NaCl, 10 mm MgCl_2_, and 10 mm DTT in 50 mm Tris‐HCl (pH 7.5 at 25 °C). For single‐molecule FRET measurements, an oxygen‐scavenging system containing 0.8% (w/w) D‐glucose (Sigma‐Aldrich), 1 mg mL^−1^ glucose oxidase (266.6 units mg^−1^, Sigma‐Aldrich), 0.4 mg mL^−1^ catalase (2000–5000 units mg^−1^, Sigma‐Aldrich) and 1 mm Trolox (Sigma‐Aldrich) was added to the reaction buffer before imaging.

### smFRET Data Acquisition and Data Analysis

The smFRET study was conducted using a homebuilt objective‐type total internal reflection fluorescence microscopy.^[^ ^25^ ^]^ Cy3 was excited by a 532 nm sapphire laser (Coherent Inc.). An oil immersion objective (100×, N.A. 1.49) was used to generate an evanescent field of illumination. The fluorescence signals from Cy3 and Cy5 were split by a dichroic mirror and then collected by an electron‐multiplying charge‐coupled‐device camera (iXON, Andor Technology). The fluorescence imaging process was controlled and recorded by MetaMorph (Molecular Devices). Prior to imaging, coverslips (Fisher Scientific) and slides were thoroughly cleaned by rinsing with acetone, methanol, a mixture of sulfuric acid and hydrogen peroxide (7:3, v/v), and sodium ethoxide. The surfaces of the coverslip were then coated with a mixture of 99% mPEG (methoxy‐PEG‐5000, Laysan Bio, Inc.) and 1% biotin‐PEG (biotin‐PEG‐5000, Laysan Bio, Inc.). An exposure time of 50 ms was used for the measurements, which were conducted at a constant temperature of 25 °C.

### Repair‐and‐Then‐Cut Experiment

In the single‐molecule experiment in Figure 2C–E, the DNA substrates were tethered on a glass surface, first treated with the T4 DNA ligase for 1 min, and then flushed with excess (>1 mL) PBS to remove ligase from the reaction chamber. Then, the nicking endonuclease Nt.BsmAI (NEB) was injected into the reaction channel to generate a new nick on the opposite strand near the original nicked site. The average number of the Cy5 spots near the Cy3 was recorded upon illumination with the 532‐nm laser is recorded.

### NS‐EM Specimen Preparation

For the T4 DNA ligase–AMP–DNA complex, T4 DNA ligase and the ddC nicked DNA were mixed (molar ratio of 1:1) in the reaction buffer and incubated for 20 mins at 25 °C. After diluting to a final concentration of 400 nm ligase and 0.3 mm ATP, the mixture was further mixed with an equal volume of 2.7 ppm (w/v) uranyl acetate (UA) and then stained, following by NS protocol as described previously, but without washing by water before staining.^[^ ^16^ ^]^ For the T4 DNA ligase–AMP complex, after incubation in the reaction buffer for 20 mins at 25 °C, the samples with a final concentration of 300 nm ligase were negatively strained by NS protocol as described.^[^ ^16^ ^]^

### EM Data Acquisition

The micrographs were collected using a FEI Talos F200C TEM equipped with a FEI CETA 16M CMOS digital camera, operating at 200 kV. With defocus values ranging from ≈0.2 to ≈0.6 µm at a magnification of 73 000× (1.607 Å/pixel), 67 micrographs for T4 DNA ligase–AMP complexes and 3765 micrographs for T4 DNA ligase–AMP–DNA complexes were collected under a dose of less than 160 e^−^/Å2 for each micrograph.

### Image Processing of EM Data

All image processing was performed within the framework of RELION‐3.0.8.^[^ ^26^ ^]^ After the contrast transfer function (CTF) parameters were determined by Gctf_v1.18,^[^ ^27^ ^]^ micrographs showing significant astigmatism or drift were excluded. For the T4 DNA ligase–AMP–DNA complex, a total of 3544 micrographs were selected for the image processing. Initially, representative class averages of manually picked particles were chosen as templates for automated particle picking. Subsequently, two different circle masks (with diameters of 120 and 160 Å) were applied for 2D classification to identify and eliminate poor‐quality particles. Following multiple iterations of reference‐free 2D classification and checking the “Einstein‐from‐noise,”^[^ ^28^ ^]^ 9057 good particles were selected and re‐centered with the presence of different DNA bending angles. Initial model maps were generated by the Relion SGD algorithm. Through multiple rounds of 3D classification, one conformation where the nicked DNA was straight (Ligase–AMP–DNA complex I, containing 3306 particles) and three conformations where the nicked DNAs were bent (Ligase–AMP–DNA complexes II, III, and IV, containing 1850, 596, and 368 particles, respectively) were identified and further refined (the reconstruction processing flowchart is illustrated in Figure S13, Supporting Information). For the T4 DNA ligase–AMP complex, after multiple iterations of 2D classification (with a mask diameter of 120 Å), 21,733 good particles were selected from a total of 67 micrographs for further processing. Four different conformations (complexes I–IV) containing 8699 particles, 3887 particles, 2598 particles, and 1465 particles were obtained and subjected to further refinement and postprocessing (the reconstruction processing flowchart is illustrated in Figure S14, Supporting Information). The resolution of each conformation was estimated by gold‐standard Fourier shell correlation 0.143 criterion.^[^ ^29^ ^]^ The method for measuring the DNA bending angles was as follows: First, we segmented the densities of DNA and ligase in each density map. Then, we identified the two DNA portions protruding at the ends of the complex and treated them as line segments. Subsequently, we measured the angle between these two lines to determine the DNA bending angles.

### Molecular Dynamics (MD) Simulations

The initial structures of T4 DNA ligase in complexes (Table S2, Supporting Information) were constructed using the MODELER module^[^ ^30^ ^]^ with crystal structure 6DT1^[^ ^9b^ ^]^ as the template. Each component system was fitted into the density map to pinpoint the spatial orientations of the ligase and DNA and subsequently refined by 100.0 ns MD simulations using GROMACS2018.8^[^ ^31^ ^]^ with Charmm36m force field.^[^ ^32^ ^]^ The topologies and parameters of AMP and its analogs were obtained from CGenFF.^[^ ^33^ ^]^ System setups for each MD simulation are listed in Table S2 (Supporting Information), consistent with the references provided.^[^ ^34^ ^]^ MD snapshots were saved every 10.0 ps for structural analysis and quantitative comparisons with EM density maps. Representative configuration of each system was chosen using the g_cluster tool implemented in GROMACS.^[^ ^35^ ^]^

### Statistical Analysis

A graphic presentation was performed using Origin 2023. Data were expressed as mean ± SD. The corresponding sample size for each statistical analysis is at least three In the single‐molecule FRET measurements, the FRET efficiency was calculated after correcting for local background, crosstalk, quantum yield, and detection efficiency,^[^ ^14^ ^]^ and an unbiased step‐finding algorithm was used to identify the FRET states in the traces.^[^ ^15^ ^]^ The data presentation and sample size for each statistical analysis were described in the corresponding figure legends. For electron microscopy image processing, all reconstructions were computed using RELION‐3.0.8, and the reconstructed 3D conformations of the ligase–AMP and ligase–DNA–AMP complexes were illustrated by UCSF Chimera.^[^ ^36^ ^]^ All molecular dynamics simulations were performed using GROMACS2018.8 with tCharmm36m force field. Structural plotting and visualization were carried out by Discovery studio client^[^ ^30a^ ^]^ and UCSF Chimera^.[^ ^36^ ^]^ The purity of the circular ssDNA, as well as the ligation efficiency, were analyzed by ImageJ.^[^ ^37^ ^]^ In brief, after background calibration, the percentage of the target band's intensity relative to the total intensity of all bands in the lane was calculated.

## Conflict of Interest

The authors declare no conflict of interest.

## Supporting information

Supporting Information

## Data Availability

The 3D EM density maps of total eight conformations have been deposited in the Electron Microscopy Data Bank under the following accession codes, respectively: EMD‐31805 (Ligase–AMP–DNA Complex I); EMD‐31747 (Ligase–AMP–DNA Complex II); EMD‐31748 (Ligase–AMP–DNA Complex III); EMD‐31749 (Ligase–AMP–DNA Complex IV); EMD‐31750 (Ligase–AMP Complex I); EMD‐31751 (Ligase–AMP Complex II); EMD‐37316 (Ligase–AMP Complex III); EMD‐31752 (Ligase–AMP Complex IV).
