# Supplementary material for: Four Parallel Pathways in T4 Ligase‐Catalyzed Repair of Nicked DNA with Diverse Bending Angles
Source: Adv Sci (Weinh). 2024 Apr 6;11(25):2401150. doi: 10.1002/advs.202401150 (PMC11220639; doi:10.1002/advs.202401150)
Supplement: Supplementary file 1 — Supporting Information [file ADVS-11-2401150-s001.pdf]

ADVANCED SCIENCE
Open Access

Supporting Information

for *Adv. Sci.*, DOI 10.1002/advs.202401150

Four Parallel Pathways in T4 Ligase-Catalyzed Repair of Nicked DNA with Diverse Bending Angles

Na Li, Jianbing Ma, Hang Fu, Zhiwei Yang, Chunhua Xu, Haihong Li, Yimin Zhao, Yizhen Zhao, Shuyu Chen, Lu Gou, Xinghua Zhang, Shengli Zhang, Ming Li, Ximiao Hou, Lei Zhang* and Ying Lu**

Supporting Information

Four Parallel Pathways in T4 Ligase-Catalyzed Repair of Nicked DNA with Diverse Bending Angles

Na Li[1,+] | Jianbing Ma[2,+] | Hang Fu[2,3,4,+] | Zhiwei Yang[1,+] | Chunhua Xu[2] | Haihong Li[5] | Yimin Zhao[1] | Yizhen Zhao[1] | Shuyu Chen[1] | Lu Gou[1] | Xinghua Zhang[6] | Shengli Zhang[1] | Ming Li[2,3,7] | Ximiao Hou[5,*] | Lei Zhang[1,*] | Ying Lu[2,3,7,*]

+ These authors contributed equally.
* Corresponding authors: houximiao@nwsuaf.edu.cn; zhangleio@mail.xjtu.edu.cn; yinglu@iphy.ac.cn.

Supporting figures

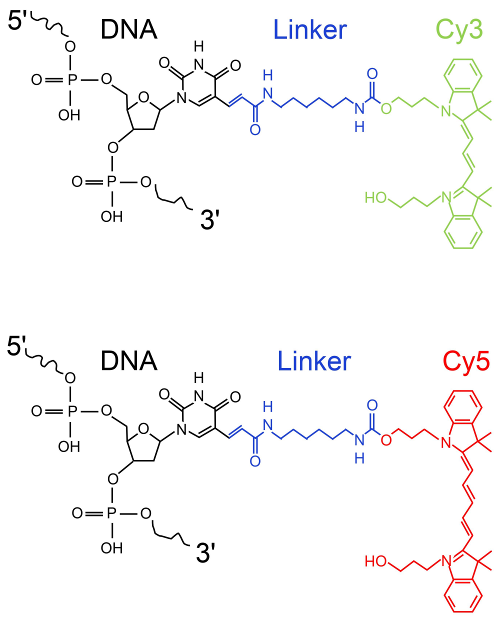

Figure S1. The structures formula of Cy3, Cy5 and the linker attaching to DNA. The DNA and the linker are in black and blue, respectively. The Cy3 and Cy5 are in green and red, respectively.

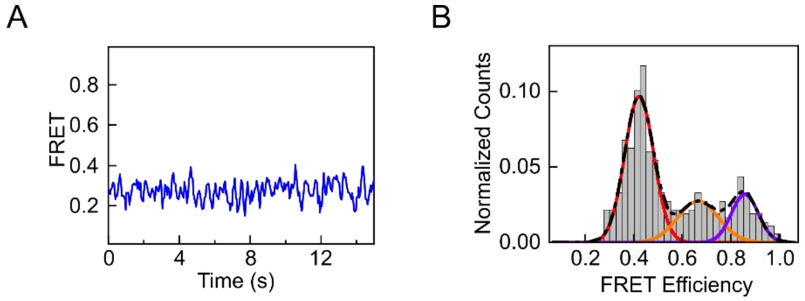

Figure S2. Typical FRET values for fluorescently labeled DNA. (A) A typical FRET trace of free DNA, with a FRET value of approximately 0.27. (B) Histogram of all high states FRET efficiency. It can be fitted with three Gaussian distributions, with the central FRET values, positions of the peaks, being 0.42, 0.67, and 0.88, respectively.

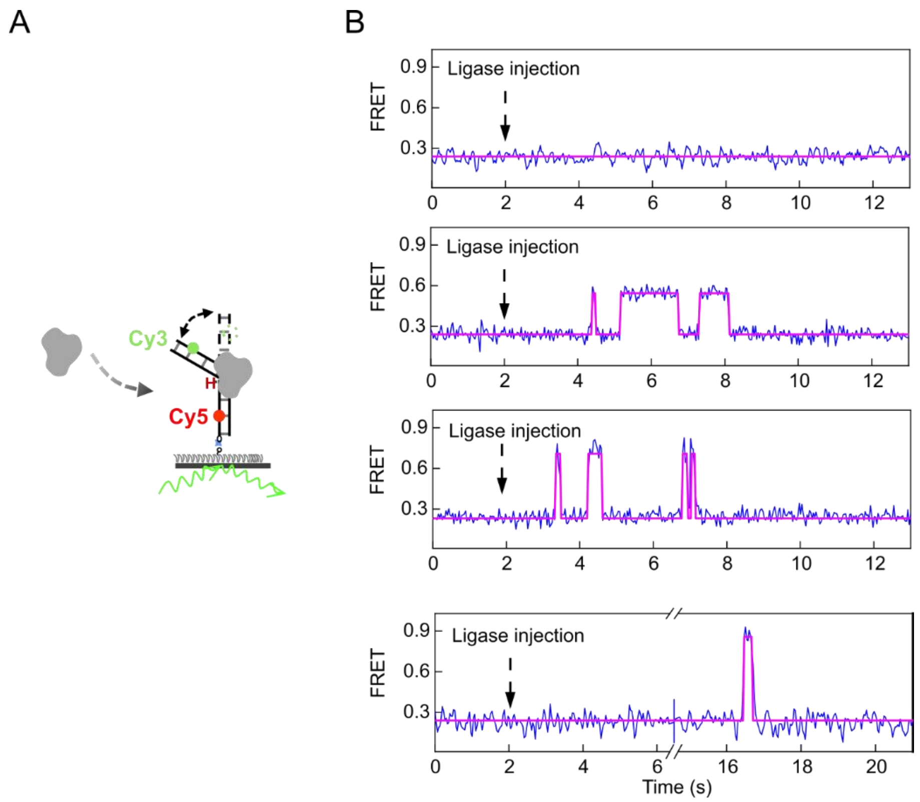

Figure S3. Conformational transitions of nonsealable nicked DNA in a ligase-AMP-DNA complex. (A) Schematic plot of the assay. Fluorescently labeled nicked DNA is immobilized on a glass surface, followed by the addition of T4 DNA ligase. Changes in fluorescence intensity are recorded in two channels, and the FRET value is calculated. The 2,3-dideoxyribonucleotide (ddC) block the final step of the nick-sealing reaction. (B) Four typical time traces of the conformational transitions.

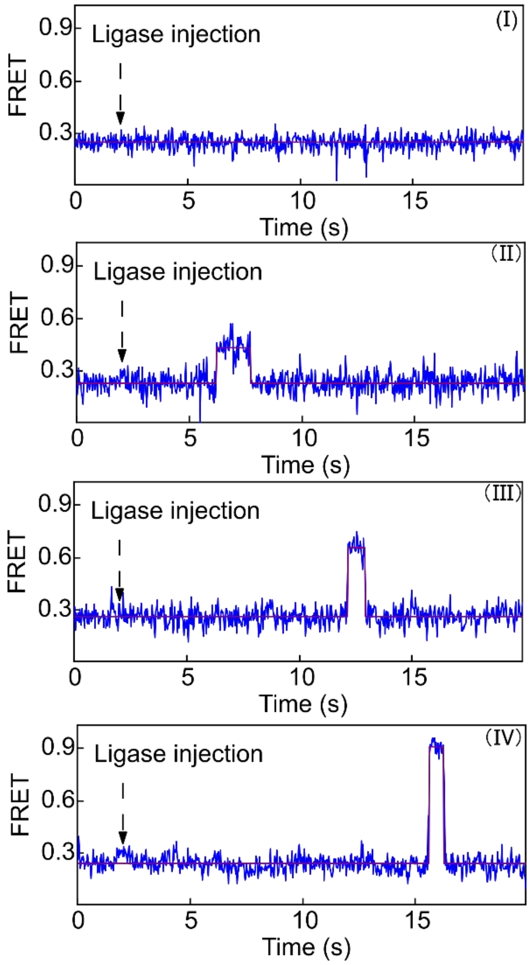

Figure S4. Conformational transitions of other sealable nicked DNA during ligation. The DNA sequence at both sides of the nick were changed in relative to the Figure 2 were tested. They also show four typical time traces of the conformational transitions.

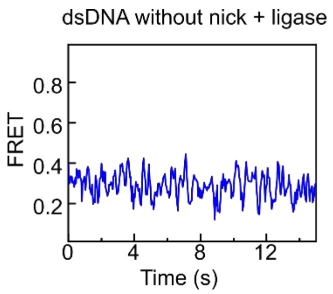

Figure S5. A typical FRET trace of dsDNA, which only shows a FRET value of approximately 0.27.

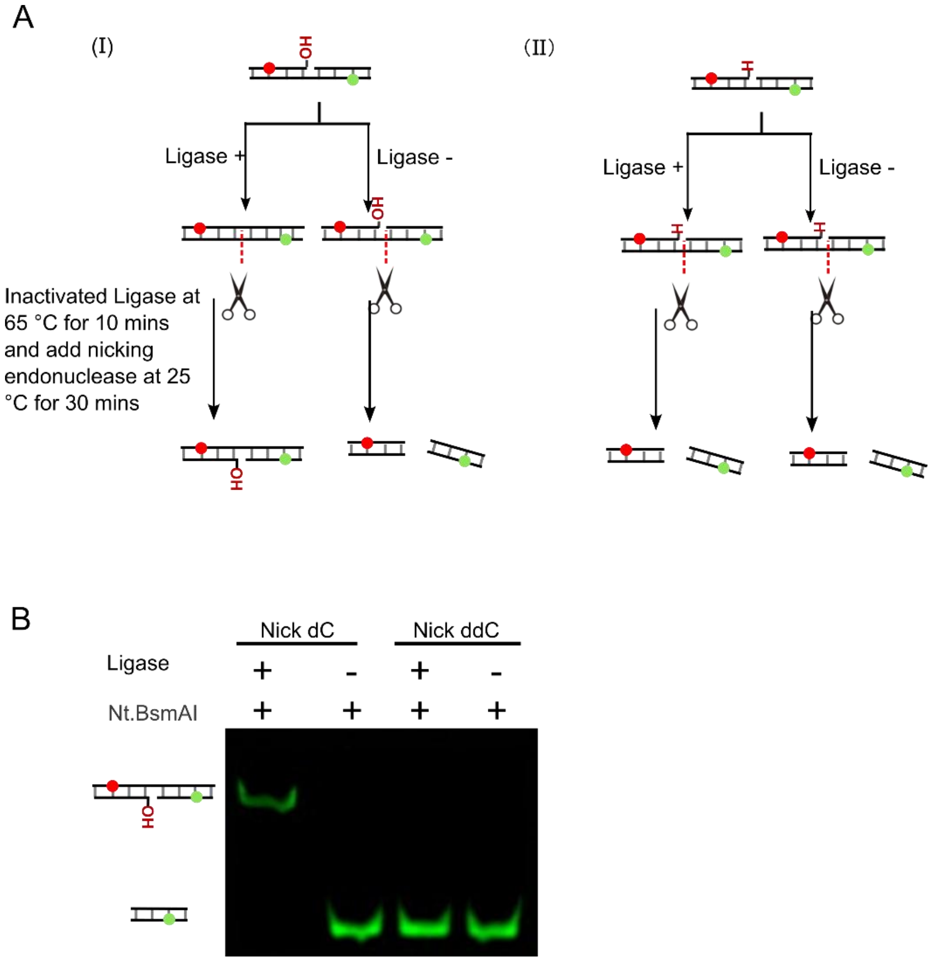

Figure S6. PAGE gel image revealed nicking endonuclease can be used to check whether T4 DNA Ligase repairs nick in DNA. (A) Schematic of cleavage product release assay for sealable nicked DNA (I) and ddC nicked DNA (II). (B) PAGE gel assay which shows the nicking endonuclease can split DNA which was not repaired by ligase.

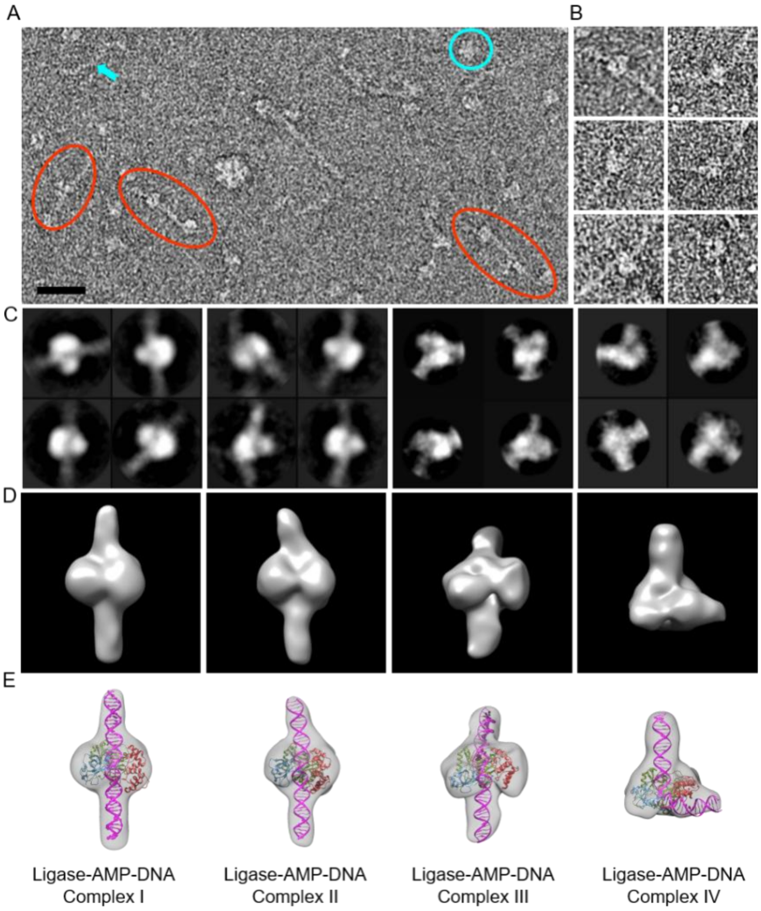

Figure S7. Reconstruction of four ligase-AMP-DNA complexes by Negative-staining electron microscope. (A) Survey views of ligase-AMP-DNA complex (in red ellipses), ligase only (in cyan circle), and DNA only (cyan arrow) are highlighted, respectively. Scale bar, 20 nm. (B) Several typical ligase-AMP-DNA complexes extracted from the raw images were presented. (C) Typical 2D reference-free class averages of the four conformations, including one straight and three bent DNA conformations. (D) 3D density maps of the four complex conformations. The maps were Gaussian low-pass filtered to 10Å. (E) The refined structural coordinates were docked into the density maps of the four ligase-AMP-DNA complex density maps. T4 DNA ligases were colored in blue for OBD, red for DBD, and green for NTase. DNAs were colored in magenta. AMPs were colored in orange.

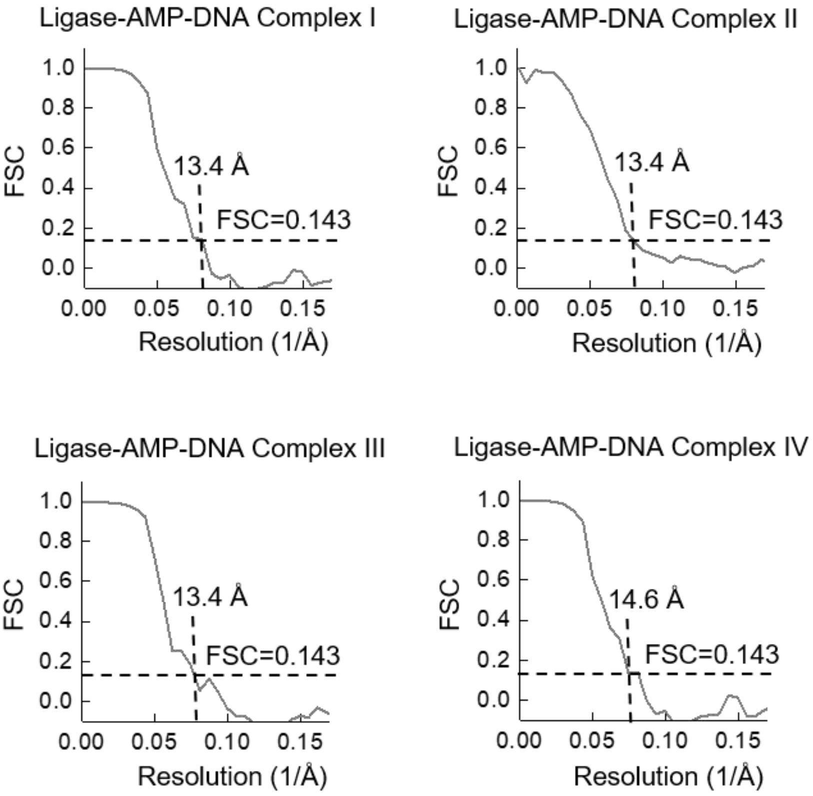

Figure S8. Fourier Shell Correction (FSC) of four ligase-AMP-DNA complex density maps.

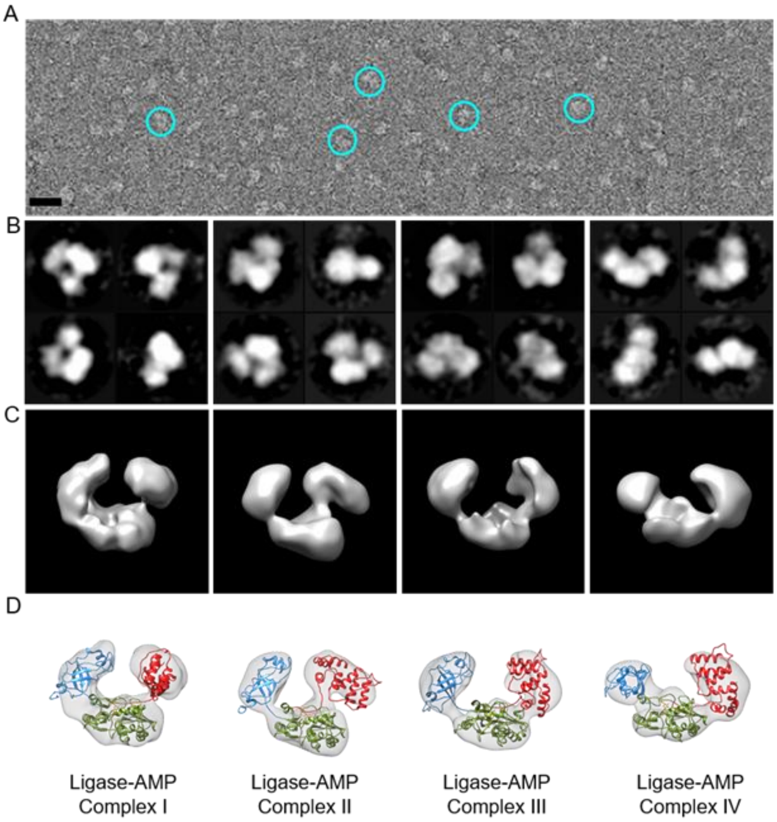

Figure S9. Four conformations of T4 DNA ligase with AMP revealed by Negative-staining TEM. (A) Survey view of T4 DNA ligase-AMP complex. Several ligases-AMP complexes are highlighted in cyan circles. Scale bar, 20 nm. (B-C) Typical 2D reference-free class averages (B), and three-dimensional density maps (C) of the four different conformations of T4 DNA ligase-AMP complex (denoted as Ligase-AMP Complex I, II, III, and IV). The maps were Gaussian low-pass filtered to 10Å. (D), The refined structural coordinates were docked into the density maps of the four conformations. T4 DNA ligases were colored in blue for OBD, red for DBD, and green for NTase. AMPs were colored in orange.

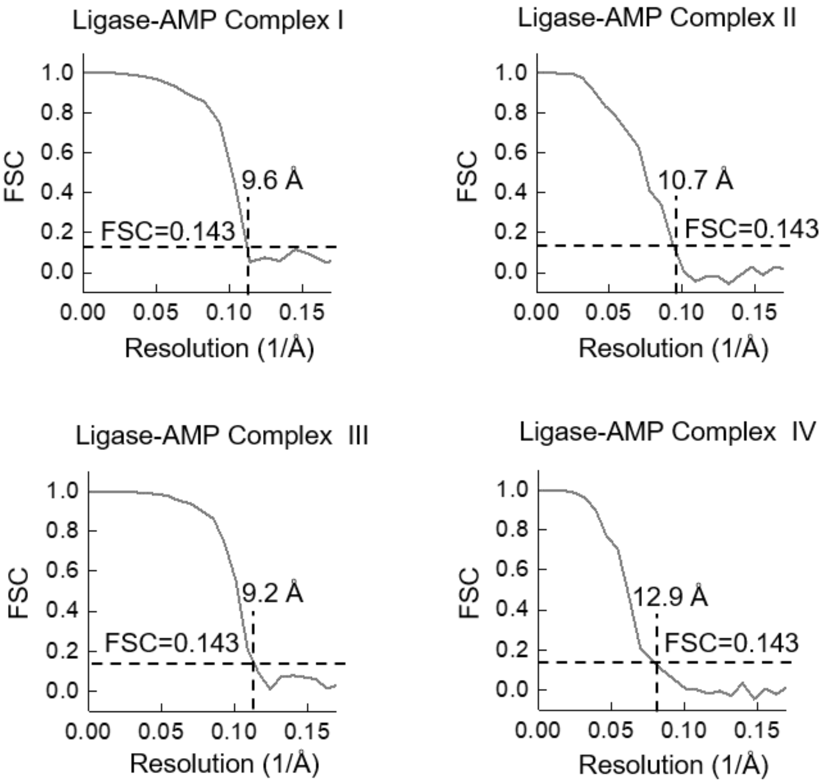

Figure S10. Fourier Shell Correction (FSC) of four ligase-AMP complex conformations density maps.

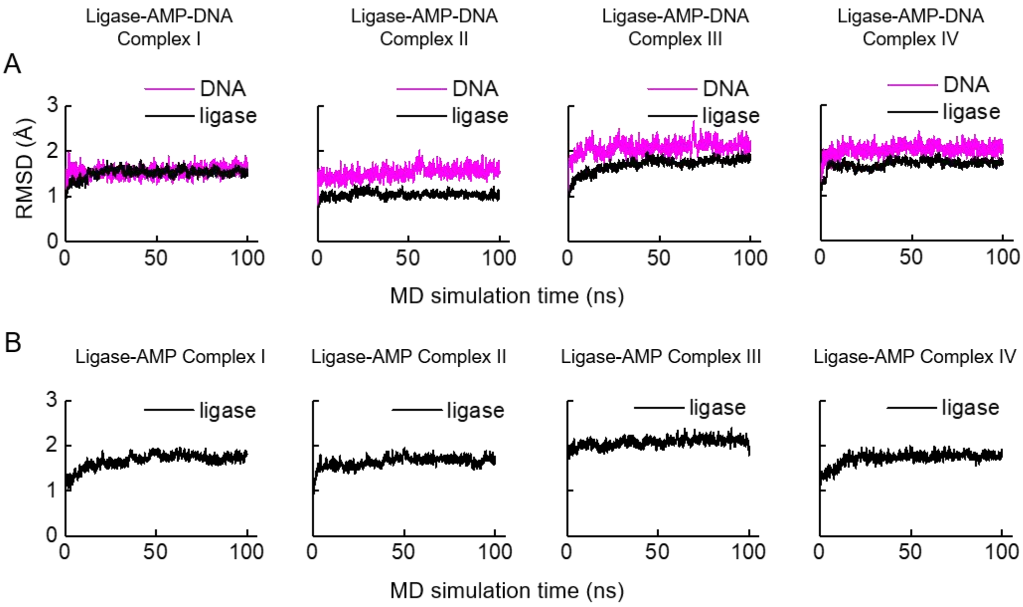

Figure S11. Time trace of backbone-atom root mean square deviations (RMSD) in the molecular dynamics simulation for the models of ligase-AMP-DNA complex conformations (A) and ligase-AMP complex conformations (B).

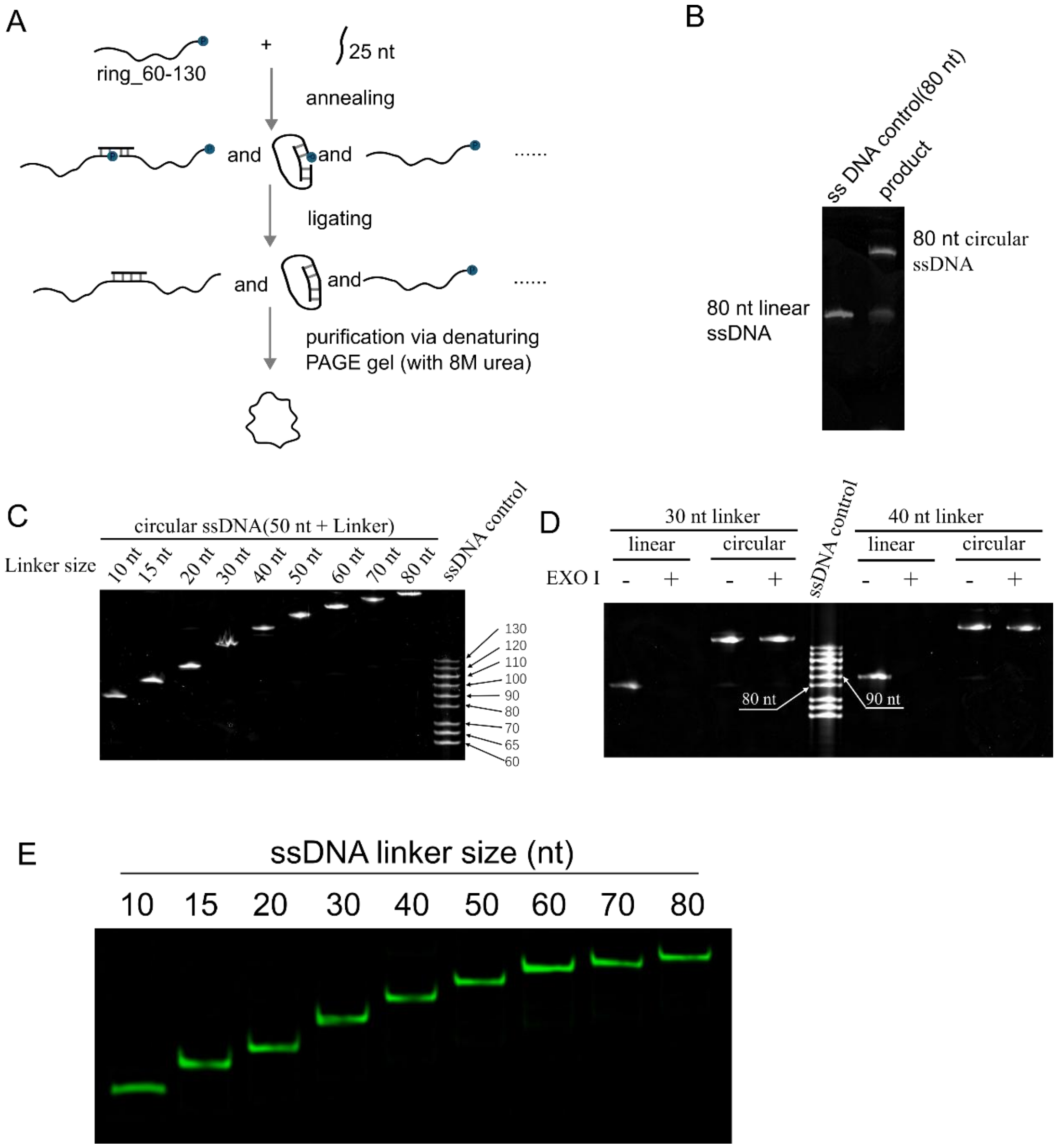

Figure S12. The preparation and verification of the DNA bow-like constructures. (A) Schematic of the synthesis process for circular ssDNA. After annealing and ligation, linear-linear ssDNA ligation, correctly circularized products, and unreacted linear ssDNA may be present. We separated the samples using 10% denaturing PAGE (with 8M urea) and isolated the DNA through ethanol precipitation. Partial digestion of linear DNA by EXO I before electrophoresis separation can be performed (optional). (B) Denaturing PAGE images(stained with GelRed) of the 80 nt ssDNA (30 nt linker) ligation products. (C) Denaturing PAGE images (stained with GelRed) of all circular DNA samples purified by electrophoresis separation and ethanol precipitation. The final purified circular ssDNA was determined to be >95% pure based on grayscale analysis. (D) Typical examination of circular ssDNA samples. Linear ssDNA is completely digested by EXO I, while circular ssDNA is resistant to digestion. The results show the examination of circular ssDNA samples with 30 nt and 40 nt linkers. Similar results were conducted for all circular samples. (E) Image of the annealed products of bow-like DNA examined using 10% PAGE (Cy3 channel).

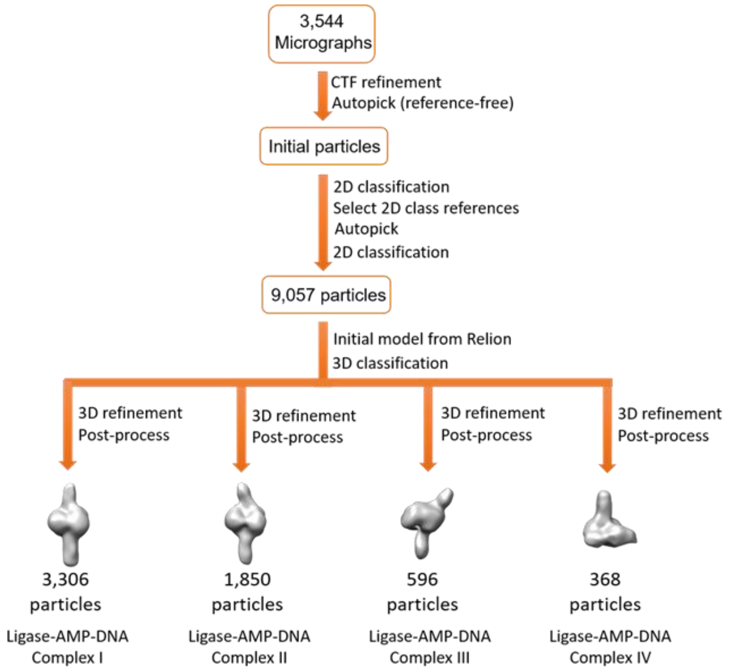

Figure S13. Negative-staining image processing workflow for ligase-AMP-DNA complex.

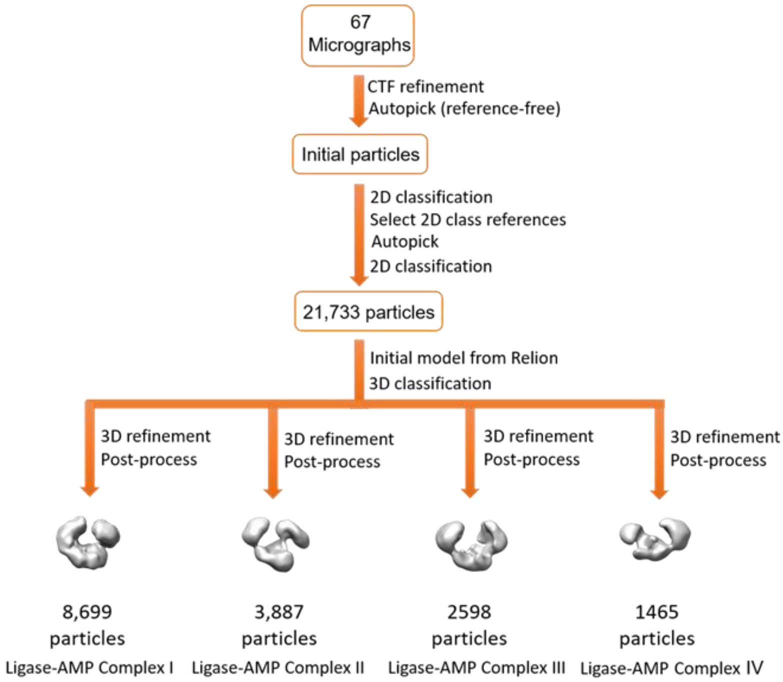

Figure S14. Negative-staining image processing workflow for ligase-AMP complex.

Supporting tables

Table S1. DNA sequences used.

Name		Sequence (5'→3')
ddC nicked DNA for FRET		**Pi**-TGCTGAGCGTATTTT(T-Cy3)ACGCTCAGCAGTTGACCATGACGTACTA
		**biotin**-TAGTACG(T-Cy5)CATGGTCAA**ddC**
Sealable nicked DNA for FRET		**Pi**-TGCTGAGCGTATTTT(T-Cy3)ACGCTCAGCAGTTGACCATGACGTACTA
		**biotin**-TAGTACG(T-Cy5)CATGGTCAAC
dsDNA for FRET		(T-cy3)ACGCTCAGCAGTTGACCATGACGTACTA
		**biotin**-TAGTACG(T-Cy5)CATGGTCAACTGCTGAGCGTA
Sealable nicked DNA for nickFRET		**Pi**-TGAGACGCGTATTTT(T-Cy3)ACGCGTCTCAGTTGACCATGACGTACTA
Change sequence for FRET		**Pi**-TGAGACGCGTATTTT(T-Cy3)ACGCGTCTCAGCCTCCGGTCACCTACTG
		**biotin**-CAGTAGG(T-Cy5)GACCGGAGGC
ddC nicked DNA for EM		GTTGACCATCTCACAGCAGTTGACCATCTCACAGCAGTTGACCATCTCACAGCAGTTGACCATCTCACAGCAGTTGACCATCTCACAGCAGTTGACCATCTCACAGCAGTTGACCATCTCACAGCAGTTGACCATCTCACAGCA
		**Pi**-TGCTGTGAGATGGTCAA**ddC**
ssDNA for circular DNA Synthesis	ring_60	**Pi-**CTACTCACCATAGCAGACCAGACCTGACGACGACTACGCTCAGCAGTTGACCATAACGTA
	ring_65	**Pi-**CTACTCACCATAGCACTGATGACCAGACCTGACGACGACTACGCTCAGCAGTTGACCATAACGTA
	ring_70	**Pi-**CTACTCACCATAGCACTCGGATGATGACCAGACCTGACGACGACTACGCTCAGCAGTTGACCATAACGTA
	ring_90	**Pi-**CTACTCACCATAGCACTCGGACCATGAGCCGACTAGTGTCATGATGACCAGACCTGACGACGACTACGCTCAGCAGTTGACCATAACGTA
	ring_100	**Pi-**CTACTCACCATAGCACTCGGACCATGAGCCGACTATTCGTGATATGTGTCATGATGACCAGACCTGACGACGACTACGCTCAGCAGTTGACCATAACGTA
	ring_110	**Pi-**CTACTCACCATAGCACTCGGACCATGAGCCGACTATTCGTGATATTCCGTCGCTGGTGTCATGATGACCAGACCTGACGACGACTACGCTCAGCAGTTGACCATAACGTA
	ring_120	**Pi-**CTACTCACCATAGCACTCGGACCATGAGCCGACTATTCGTGATATTCCGTCGCTGCTGGCGCTGGGTGTCATGATGACCAGACCTGACGACGACTACGCTCAGCAGTTGACCATAACGTA
	ring_130	**Pi-**CTACTCACCATAGCACTCGGACCATGAGCCGACTATTCGTGATATTCCGTCGCTGCTGGCGCTGGCCTCGTGGTAGTGTCATGATGACCAGACCTGACGACGACTACGCTCAGCAGTTGACCATAACGTA
ssDNA for broken linker DNA Synthesis	linear_50	GACCTGACGACGACTACGCTCAGCAGTTGACCATAACGTACTACTCACCA
	linear_130	CGCTGCTGGCGCTGGCCTCGTGGTAGTGTCATGATGACCAGACCTGACGACGACTACGCTCAGCAGTTGACCATAACGTACTACTCACCATAGCACTCGGACCATGAGCCGACTATTCGTGATATTCCGT
25 nt Oligo	Oligo_1	TGGTGAGTAGTACGTTA(T-Cy3)GGTCAAC
	Oligo_2	**Pi**-TGCTGAGCGTAGTCGTCGTCAGGTC

Table S2. Summary of each simulation and corresponding MD time scale.

Simulation	System setup	Time (ns)
Ligase-AMP-DNA Complex I	T4 DNA ligase + AMP + nick DNA (bend ~0°)	100
Ligase-AMP-DNA Complex II	T4 DNA ligase + AMP + nick DNA (bend ~20°)	100
Ligase-AMP-DNA Complex III	T4 DNA ligase + AMP + nick DNA (bend ~60°)	100
Ligase-AMP-DNA Complex IV	T4 DNA ligase + AMP + nick DNA (bend ~100°)	100
Ligase-AMP Complex I	T4 DNA ligase + AMP	100
Ligase-AMP Complex II	T4 DNA ligase + AMP	100
Ligase-AMP Complex III	T4 DNA ligase + AMP	100
Ligase-AMP Complex IV	T4 DNA ligase + AMP	100

[a)] All simulations include SPC/E water box, which extends at least 10.0 Å from any solute atom.
